# A rare case of advanced sinonasal adenoid cystic carcinoma with intracranial and intradural extension

**DOI:** 10.1093/jscr/rjac038

**Published:** 2022-02-17

**Authors:** Joseph Rassam, Tarun Sood

**Affiliations:** Department of Ear, Nose and Throat Surgery, Leicester Royal Infirmary, Leicester, UK

## Abstract

Adenoid cystic carcinoma (ACC) is a rare cancer of the head and neck that primarily occurs in the salivary glands. Sino-nasal ACC (SNACC) is a much rarer entity; this paper presents an exceedingly rare case of SNACC with both intracranial and intradural extension which was ultimately treated with palliative radiotherapy due to its extensive invasion. In addition to this, a review of the literature has been performed to delineate specific learning points for the management of intracranial SNACC.

## INTRODUCTION

Adenoid cystic carcinoma (ACC) is a rare cancer of the head and neck that primarily occurs in the salivary glands. ACC is not exclusive to the head and neck and can seldom arise in various locations such as the respiratory tract, oesophagus, breast and genitourinary system. Histologically, it originates from pseudostratified columnar epithelium. It is characterized by slow growth and has a high tendency for perineural invasion and local recurrence [1]. The overall 5-year survival is 60–80% and the single most important prognostic factor is the presence or absence of distant metastasis (DM) [2–6].

Sino-nasal ACC (SNACC) is a much rarer entity; this paper presents a case of SNACC with intracranial and intradural extension and performs a review of literature.

## CASE

A female aged in her 50s was referred to the ENT and ophthalmology team by her general practitioner with 6 weeks of persistent nasal obstruction, purulent nasal discharge, loss of smell/taste, swelling of the eye and blurred vision. She had a past medical history of hypertension, asthma, uterine fibroadenoma and a significant smoking history.

**Figure 1:**
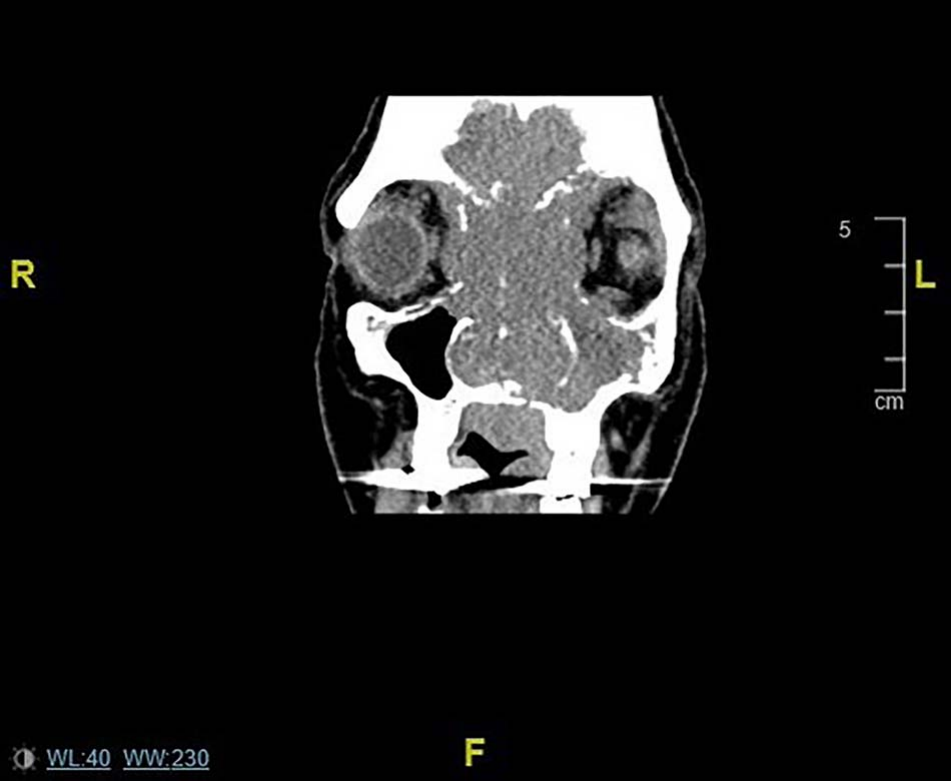
Coronal CT sinuses/head images displaying sinonasal mass invading anterior skull base, orbits and intracranially.

Initially she was assessed by the ophthalmology team and seen to have congestion of the right eye associated with nasal obstruction and left maxillary swelling. When subsequently seen by ENT she was referred for urgent computed tomography (CT) sinuses and review in the head and neck cancer clinic. CT sinuses performed displayed a large malignant mass in the nasal cavity extending to the paranasal sinuses and invading the facial skeleton and anterior skull base (Fig. 1).

**Figure 2:**
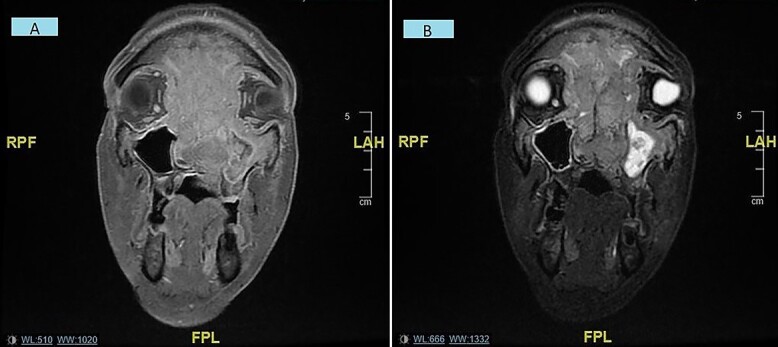
(**A** and **B**) Coronal MRI sinuses/head images displaying sinonasal mass invading anterior skull base, orbits and intracranially/intradurally (2A—T1 weighted, 2B—STIR).

A subsequent magnetic resonance imaging (MRI) was performed within 2 weeks at which point there was complete visual loss. This displayed a large malignant soft tissue mass extending through the nasal cavity with superior intracranial extension plus optic nerve compression and bilateral intra-orbital extension (Fig. 2). A staging CT displayed no DM. An EUA nose plus biopsy was performed, which exhibited extensive invasion in the nasal cavity bilaterally.

Histology revealed respiratory mucosa with diffuse infiltration of tumour composed of enlarged cells with hyperchromatic nuclei and scanty cytoplasm with cribriform and glandular architecture—suggestive of either ACC- or HPV-related sino-nasal carcinoma. With further immunohistochemistry a diagnosis of SNACC was confirmed.

Six weeks from the initial presentation, this case was discussed at the multidisciplinary team meeting (MDT) with all the radiological and histological information necessary; it was decided that palliative radiotherapy would be the best course of action.

She commenced radiotherapy 2 weeks after the MDT meeting but unfortunately was admitted to hospital 3 days following her first dose with systemic issues i.e. pneumonia, electrolyte derangements, acute kidney injury and thrombocytopenia. She was provided best supportive care with palliative care input. She died at home 2 days following discharge.

## DISCUSSION

Most sino-nasal carcinomas arise in the maxillary sinuses and are primarily squamous cell carcinomas, although adenocarcinomas are described, especially in woodworkers [7, 8]. ACC is commonly found in salivary glands and much more rarely in the sino-nasal region [4].

Due to inherent bone involvement, initial treatment is usually surgical, with consideration for adjuvant radiation therapy based upon stage and pathologic findings. Reconstruction and rehabilitation, especially in cases with orbital involvement, may be prosthetic or tissue based. Sino-nasal carcinomas of the anterior skull base include a variety of pathologies. Standard treatment is multidisciplinary, including craniofacial surgical intervention with adjuvant radiation with or without chemotherapy. Charged- particle radiation, such as proton beam radiation, may be considered in patients with involvement near the anterior skull base and/or orbit [9].

There are few larger studies of SNACC. A meta-analysis, systematic review and large retrospective study of SNACC highlight that it commonly presents as a T3–4 tumour and has the best prognosis when surgical resection is feasible. The estimated 5-year survival from aggregated data is 62% [3, 10, 11]. Evidence with regards to efficacy of adjuvant radiotherapy in SNACC is conflicting; meta-analysis from Amit *et al*. [10] shows no prognostic benefit, while systematic review from Husain et al. [11] suggests some benefit of post-operative radiotherapy.

Intracranial extension of SNACC is extremely rare and only described by isolated case reports. The management of intracranial SNACC is largely dependent on whether intradural invasion is present; intradural spread is a contraindication for surgical resection due to the significant neurological risk.

Benazzou *et al*. [12] and Veillon *et al*. [13] both describe cases of SNACC with intracranial extension but without intradural invasion successfully treated with surgical resection and adjuvant radiotherapy. Giridhar *et al*. [14] describe a case of SNACC which was inoperable as had invaded the cavernous sinus. Interestingly, this showed complete resolution after 6 months of radical radiotherapy, which contradicts the understanding that ACC has relatively low radiosensitivity. Sepúlveda *et al*. [15] describe a case of SNACC with intradural extension which was managed with palliative chemo-radiotherapy.

## CONCLUSIONS

The rarity of SNACC with intracranial extension makes it hard to standardize treatment of these patients. Feasibility of surgery is largely dependent on the presence or absence of intradural invasion which subsequently impacts prognosis. If intradural invasion is present, the mainstay of treatment will be palliative (primarily radiotherapy).

Surgical resection in our case was not feasible due to the extensive intradural invasion. Palliative radiotherapy was offered as supportive measure to reduce symptomatic burden. There should be a focus on early diagnosis to maximize the proportion of patients who are eligible for surgical treatment.

## CONFLICT OF INTEREST STATEMENT

None declared.
